# Supplementary material for: Molecular principles underlying dual RNA specificity in the Drosophila SNF protein
Source: Nat Commun. 2018 Jun 7;9:2220. doi: 10.1038/s41467-018-04561-6 (PMC5992148; doi:10.1038/s41467-018-04561-6)
Supplement: Supplementary file 1 — Supplementary Information [file 41467_2018_4561_MOESM1_ESM.pdf]

Molecular principles underlying dual RNA specificity in the *Drosophila* SNF protein

Weber *et al.*

Supplementary Figures

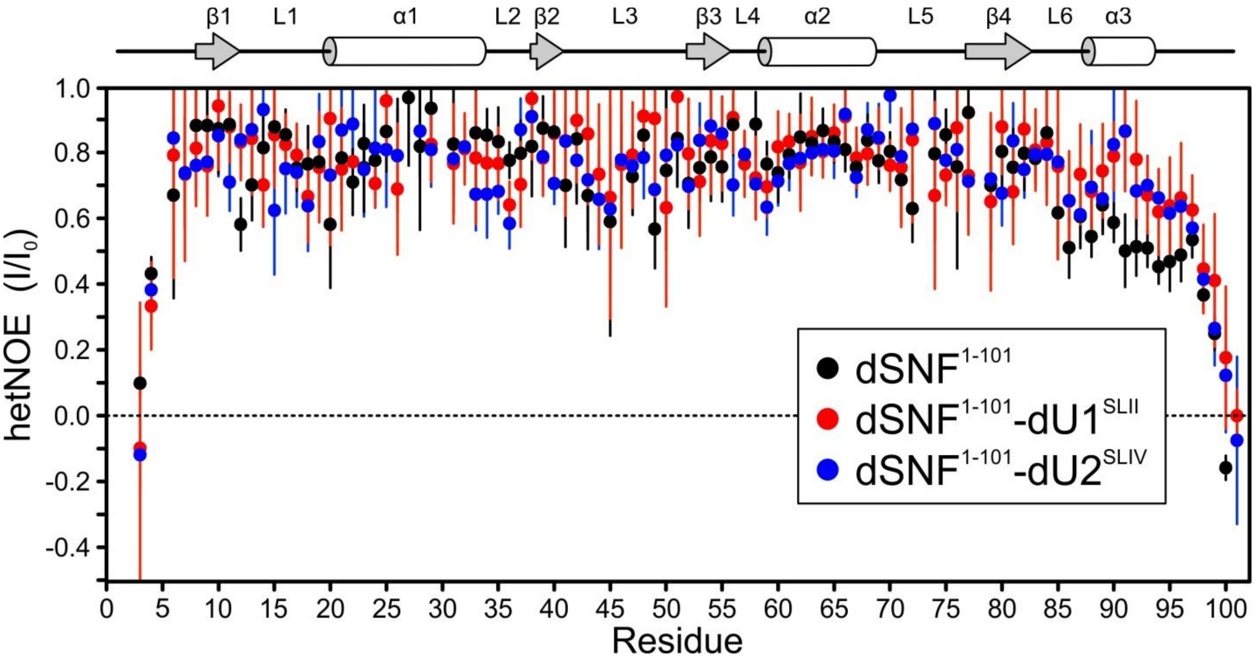

Supplementary Figure 1. NMR ^{15}N/^{1}H heteronuclear NOEs.

NMR ^{15}N/^{1}H heteronuclear NOEs of dSNF$^{1\text{-}101}$, dSNF$^{1\text{-}101}$-dU1^{SLII} and dSNF$^{1\text{-}101}$-dU2^{SLIV}. ^{15}N/^{1}H heteronuclear NOE reports rapid (ps-ns) dynamics of backbone amides. Values < 0.6 indicate significant motion, seen here for the N-terminal and C-terminal residues of free dSNF$^{1\text{-}101}$. Addition of RNA reduces dynamics of helix α3. Heteronuclear TROSY ^{15}N-{^{1}H} NOE data were acquired at 300 µM dSNF$^{1\text{-}101}$, 300 µM dSNF$^{1\text{-}101}$:330 µM dU1^{SLII} and 300 µM dSNF$^{1\text{-}101}$:600 µM dU2^{SLIV}. ● - dSNF$^{1\text{-}101}$; ● - dSNF$^{1\text{-}101}$-dU1^{SLII}; ● - dSNF$^{1\text{-}101}$-dU2^{SLIV}. Data are averages of three pairs of on(I)/off(I_o) resonance experiments. Errors were determined form the propagation of base plane rms noise. 50 mM KCl, 20 mM sodium cacodylate, pH 6.5, in 90 % $^{1}H_2O$, 10 % $^{2}H_2O$; 23° C.

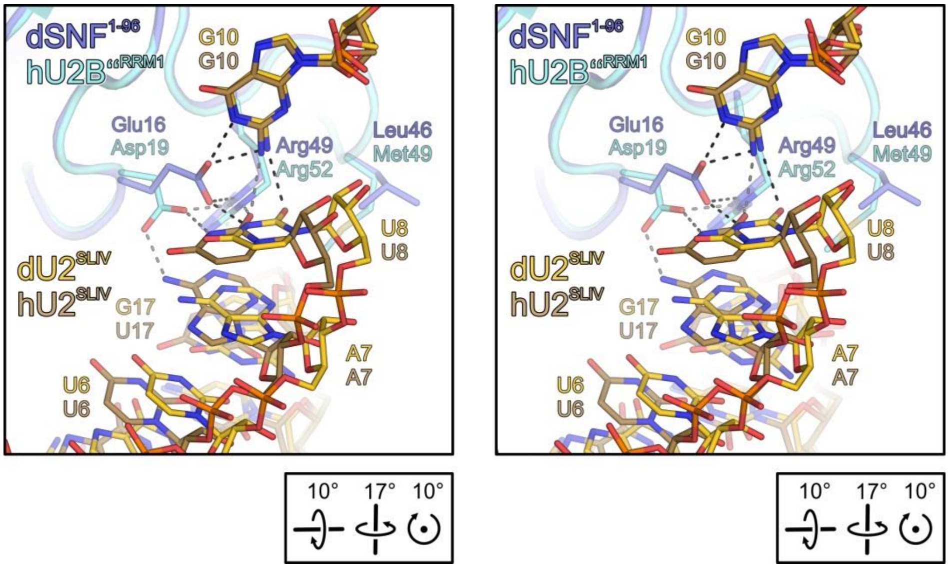

Supplementary Figure 2. dSNF$^{1\text{-}96}$-dU2^{SLIV} and hU2B''^{RRM1}-hU2^{SLIV} interactions.

hU2A'-hU2B''^{RRM1}-hU2^{SLIV} complex (fern green/cyan/light brown; PDB ID 1A9N [14]) superimposed on the dU2A'-dSNF$^{1\text{-}96}$-dU2^{SLIV} complex (green/steel blue/gold) according to the hU2B''^{RRM1}/dSNF$^{1\text{-}96}$ subunits.

Supplementary Tables

Supplementary Table 1. Crystallographic data and refinement[a]

	dSNF$^{1\text{-}96}$	dSNF$^{1\text{-}96}$-dU1^{SLII}	dU2A'-dSNF$^{1\text{-}96}$	dU2A'-dSNF$^{1\text{-}96}$-dU2^{SLIV}
**Data collection**				
**Wavelength** (Å)	0.9184	0.9184	0.9184	0.9184
**Temperature** (K)	100	100	100	100
**Space group**	$P2_12_12_1$	$C222_1$	P2	$P2_12_12_1$
**Unit cell parameters**<br>a/b/c (Å)<br>α/ß/γ (°)	 52.3/119.7/122.8<br>90/90/90	 56.7/150.0/133.6<br>90/90/90	 66/53.7/75.8<br>90/103.1/90	 65/103.9/116.3<br>90/90/90
**Resolution** (Å)	50 - 1.49<br>(1.58 -1.49)	50 - 2.00<br>(2.12 - 2.00)	50 - 1.42<br>(1.50 - 1.42)	50 - 1.90<br>(2.01 - 1.90)
**Reflections**<br>Unique<br>Completeness (%)<br>Redundancy	 125,106 (5,057)<br>98.6 (98.4)<br>4.5 (4.5)	 39,021 (6,164)<br>99.7 (99.1)<br>10.9 (10.7)	 97,210 (15,154)<br>99.0 (96.1)<br>3.6 (3.26)	 62,952 (9,932)<br>99.7 (98.5)<br>5.78 (5.74)
**I/σ(I)**	22.1 (1.9)	15.8 (1.8)	10.3 (1.5)	11.64 (1.68)
**R_{sym}(I)**[b]	0.03 (0.94)	0.11 (1.22)	0.07 (0.78)	0.14 (1.20)
**$CC_{1/2}$**[c]	100 (77.8)	99.9 (66.2)	99.8 (63.6)	99.7 (57.5)
**Refinement**				
**Resolution** (Å)	24.32-1.49	38.3 - 2.0	19.90 - 1.42	47.4 – 1.9
**Reflections**<br>Number<br>Completeness (%)<br>Test set (%)	 125,046<br>98.6<br>5.0	 39,017<br>99.7<br>5.0	 97,179<br>99.0<br>5.0	 62,935<br>99.7<br>3.3
**R_{work}**[d]	0.146	0.169	0.146	0.167
**R_{free}**[d]	0.188	0.212	0.193	0.218
**ESU** (Å)[e]	0.15	0.26	0.15	0.22
**Contents of A.U.**[f]<br>RNA/residues<br>Proteins/residues<br>Water oxygens	 -<br>6/528<br>630	 3/66<br>3/297<br>378	 -<br>4/533<br>665	 2/50<br>4/535<br>883
**Mean B-factors**<br>Wilson (Å^2)<br>Protein (Å^2)<br>Solvent (Å^2)	 22.6<br>35.1<br>46.1	 34.8<br>48.6<br>43.9	 14.8<br>23.9<br>35.6	 24.0<br>33.1<br>39.2
**Ramachandran plot**[g]<br>Favored (%)<br>Allowed (%)<br>Outliers (%)	 99.06<br>0.94<br>-	 98.91<br>1.09<br>-	 97.48<br>2.52<br>-	 97.29<br>2.53<br>0.18
**Rmsd**[h]<br>Bond lengths (Å)<br>Bond angles (°)<br>Dihedral angles (°)	 0.016<br>1.520<br>16.4	 0.012<br>1.259<br>15.5	 0.007<br>1.183<br>14.1	 0.015<br>1.389<br>14.5

[a] Data for the highest resolution shell in parentheses.

[b] $R_{sym}(I) = \Sigma_{hkl}\Sigma_i |I_i(hkl) - \langle I(hkl)\rangle| / \Sigma_{hkl}\Sigma_i |I_i(hkl)|$; for n independent reflections and i observations of a given reflection; $\langle I(hkl)\rangle$ – average intensity of the i observations.

[c] $CC_{1/2}$, correlation factor between random half-datasets as evaluated by XSCALE[1].

[d] $R = \Sigma_{hkl} ||F_{obs}| - |F_{calc}|| / \Sigma_{hkl} |F_{obs}|$; R_{work} – hkl $\notin$ T; R_{free} – hkl $\in$ T; T – test set (5 %).

[e] ESU – estimated overall coordinate error based on maximum likelihood.

[f] A.U. – asymmetric unit.

[g] Calculated with MolProbity (http://molprobity.biochem.duke.edu/)[2].

[h] Rmsd – root-mean-square deviation from target geometry.

Supplementary Table 2. DNA oligonucleotides used in this study

Name	Sequence
SNF_NcoI_f	TATCCATGGAGATGCTACCCAACC
SNF_96_Xho1_r	GTACTCGAGTTAACCCTTTATCTTGGCCACAATATC
SNF_COEX_96_r	CCTTCTTAAAGTTAAACAAAATTATTTCTAGATGATCTTTAACCCTTTATCTTGGCCACAATATCCG
U2A_COEX_f	GAAATAATTTTGTTTAACTTTAAGAAGGAGATATACATATGGTGAAACTAACGCCGGAGCTG
U2A_176_XhoI_r	GTACTCGAGTTACCGGCTAATCTCCTTTAGCACGTCC
U2A'R20A_f	CGATGCAATACATAAACCCAGTCGCGGAGCGCGAGCTGGATTTGCGCGGC
U2A'R20A_r	GCCGCGCAAATCCAGCTCGCGCTCCGCGACTGGGTTTATGTATTGCATCG
U2A'R143AK149A_f	GGCCTACAAGTTCCCGCAGCTGGCCCTACTCGACTTCAGGGCCATCAAGCAGAAGGACCGCCAGGCGG
U2A'R143AK149A_r	CCGCCTGGCGGTCCTTCTGCTTGATGGCCCTGAAGTCGAGTAGGGCCAGCTGCGGGAACTTGTAGGCC
U2A’KQK151-153AAA_f	CTTCAGGGCCATCGCAGCCGCGGACCGCCAGGCGG
U2A’KQK151-153AAA_r	CCGCCTGGCGGTCCGCGGCTGCGATGGCCCTGAAG
SNF_K17A_f	CAATCTGAACGAGGCGATCAAGAAGGAGG
SNF_K17A_r	CCTCCTTCTTGATCGCCTCGTTCAGATTG
T7_promoter	TAATACGACTCACTATAG
T7_Wt_U2_SLIV	GGACCCGGCGGTACTGCAATACCGGCCTATAGTGAGTCGTATTA
T7_Wt_U1_SLII	GGACCCGGCGCGTGGTGCAATGCCGGCCTATAGTGAGTCGTATTA

Supplementary References

1. Kabsch, W. XDS. *Acta Crystallogr. D* **66,** 125–32 (2010).

2. Chen, V. B. *et al.* MolProbity: all-atom structure validation for macromolecular crystallography. *Acta Crystallogr. D* **66,** 12–21 (2010).
